# A Lesion-adaptive Segmentation Approach for Tumor Delineation on FDG PET/CT in Diffuse Large B-cell Lymphoma Patients

**DOI:** 10.1007/s00259-026-07768-8

**Published:** 2026-02-14

**Authors:** Gerben J. C. Zwezerijnen, Martijn W. Heymans, Danielle van Assema, Michael D. A. van Elk, Jakoba J. Eertink, Patricia C. Spa, Sanne E. Wiegers, Pieternella J. Lugtenburg, Yvonne W. S. Jauw, Otto S. Hoekstra, Josée M. Zijlstra, Ronald Boellaard

**Affiliations:** 1 Department of Radiology and Nuclear Medicine, Amsterdam UMC, Vrije Universiteit Amsterdam, de Boelelaan 1117, Amsterdam, The Netherlands; 2 Cancer Center Amsterdam, Imaging and Biomarkers, Amsterdam, The Netherlands; 3 Department of Epidemiology and Data Science, Amsterdam UMC location Vrije Universiteit Amsterdam, De Boelelaan 1117, Amsterdam, The Netherlands; 4 Amsterdam Public Health Research Institute, Methodology, Amsterdam, The Netherlands; 5 Department of Medical Imaging, Nuclear Medicine, Northwest Clinics, Alkmaar, The Netherlands; 6 Department of Hematology, Erasmus MC Cancer Institute, University Medical Center, Rotterdam, The Netherlands; 7 Department of Hematology, Amsterdam UMC, Vrije Universiteit Amsterdam, De Boelelaan 1117, Amsterdam, The Netherlands

**Keywords:** Diffuse large b-cell lymphoma, FDG PET/CT, Metabolic tumor volume, Segmentation method, Lesion-adaptive delineation

## Abstract

**Purpose:**

SUV4.0-based thresholding is widely used for baseline [¹⁸F]FDG PET-based metabolic tumor volume (MTV) assessment in diffuse large B-cell lymphoma (DLBCL), but its suitability at interim and end-of-treatment (EoT) PET, when residual uptake is heterogeneous and tumor-to-background contrast is lower, is uncertain. We aimed to define a lesion-adaptive decision rule approach for selecting the optimal segmentation method based on lesion-level features and treatment phase and, exploratorily, to compare its performance with ML-based selection models.

**Methods:**

A total of 598 lesions from 33 DLBCL patients (HOVON-84 trial) were segmented at baseline, interim, and EoT [¹⁸F]FDG PET/CT using six semi-automated methods: SUV2.5, SUV4.0, 41%max, A50peak, MV2, and MV3. Segmentation quality was independently rated for each lesion by two observers (scale 1–5; 3 = preferred), with adjudication by a third reviewer. The influence of lesional SUVpeak, tumor-to-background ratio (TBRpeak), background uptake (SUVbg), treatment phase, and location on segmentation quality was assessed. Over six million rule-based combinations of key features were evaluated to derive a lesion-adaptive decision rule for preferred method selection. Exploratorily, ML classifiers were trained and compared with the decision-rule strategy.

**Results:**

Segmentation quality varied across lesions and methods. SUVpeak, TBRpeak, and SUVbg were key predictors of method performance. The final lesion-adaptive rule, applying SUV4.0 if SUVpeak > 8, MV3 if SUVbg > 0.8, and otherwise MV2, achieved a lesion-wise accuracy of 0.82 for preferred method selection, matching the best-performing ML models. Versus SUV4.0 alone (benchmark), the Decision Rule improved lesion-level MTV agreement with the reference (ρ = 0.85 vs. 0.82 vs. best ML ρ = 0.81) and reduced the proportion of lesions with > 10% MTV deviation (46.2% vs. 63.5%; best ML 50.2%). Total-MTV agreements with the reference were uniformly high across all strategies (all ρ ≥ 0.94), with modest gains for the decision rule at interim and EoT PET.

**Conclusion:**

A straightforward decision-rule approach using SUVpeak and SUVbg successfully selects the preferred method for individual DLBCL lesions across treatment phases and matches ML performance with greater simplicity and clinical applicability. Although supervision remains necessary, this approach helps address the current gap in segmentation methodology for interim and EoT PET, where SUV4.0 may not always be appropriate.

**Graphical Abstract:**

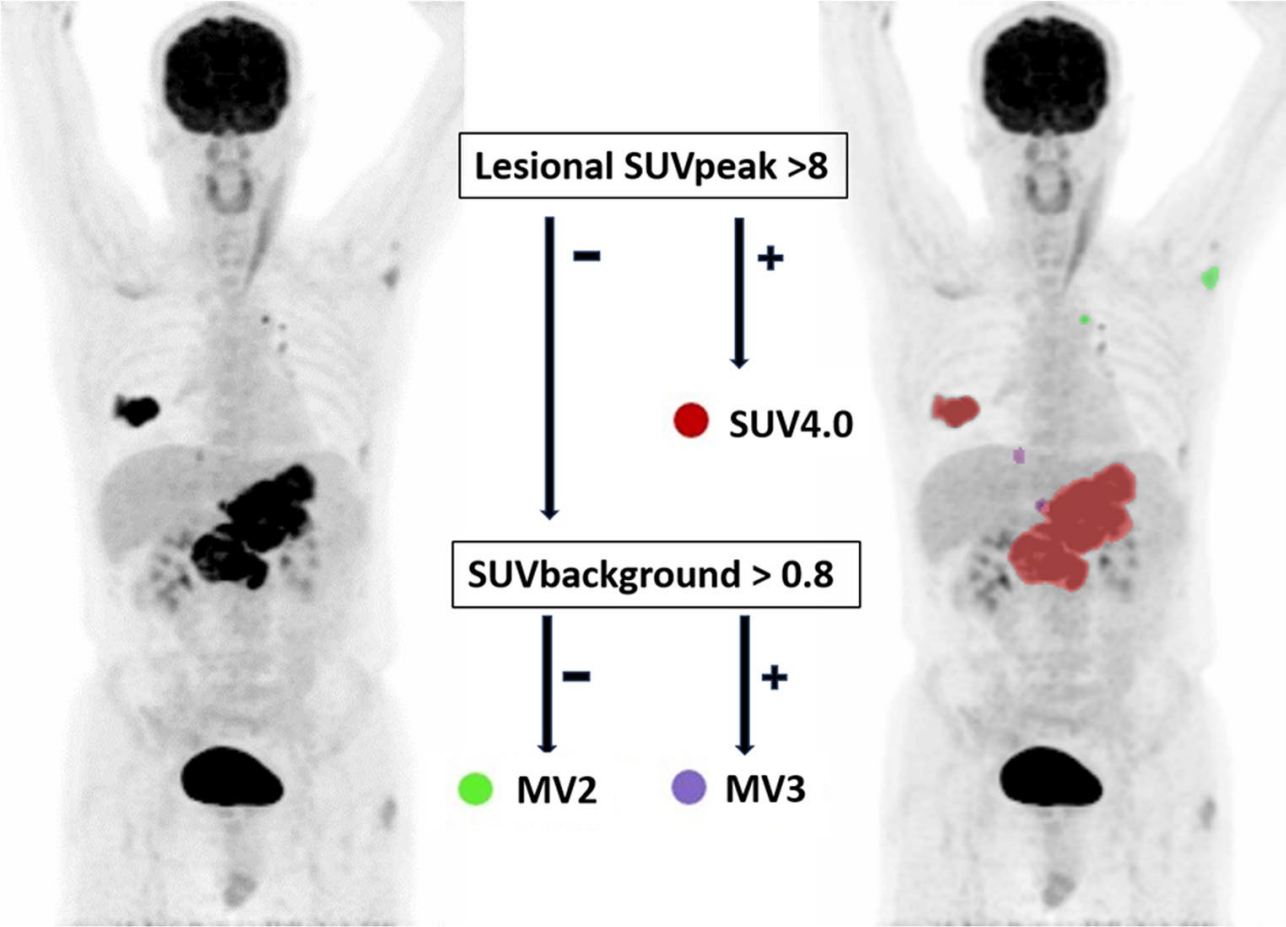

**Supplementary Information:**

The online version contains supplementary material available at 10.1007/s00259-026-07768-8.

## Introduction

Diffuse large B-cell lymphoma (DLBCL), the most prevalent subtype of non-Hodgkin lymphoma (NHL), is characterized by significant clinical and biological heterogeneity, which manifests in highly variable metabolic activity and distribution of (extra)nodal lesions among patients [1]. This large heterogeneity in genotype and immunophenotype poses challenges in prognosis and the evaluation of treatment outcomes [1, 2]. 

Metabolic tumor volume (MTV), a quantitative parameter derived from ^18^F-FDG positron emission tomography (PET), offers valuable, if still partial, insights into the glycolytic phenotype and tumor burden in DLBCL. Numerous studies have consistently shown that MTV contributes to estimating prognosis and predicting treatment response, albeit acknowledging that different segmentation methods yielded varying MTV cutoffs [3–8]. The International Metabolic Prognostic Index (IMPI), which enables individualized estimates of patient outcomes, has further highlighted the significance of baseline MTV and its applicability to risk-adapted treatment strategies in clinical trials [9]. Additionally, preliminary data suggest the potential of MTV at interim- (I-) and end-of-treatment (EoT) PET [7, 10–12].

Several semi- and fully automatic segmentation algorithms for assessing MTV have been developed. Among these, SUV4.0 seems to be most successful at baseline PET, based on interobserver reliability and ease of use, and is, therefore recently proposed as the benchmark segmentation method in DLBCL at baseline [4, 6, 9, 13]. However, delineation quality and reproducibility of MTVs using threshold SUV4.0 or other fixed, relative, or gradient thresholds at interim and EoT PET are questionable compared to baseline PET [14]. Specifically, the frequently low lesion-background contrast and pronounced heterogeneity in metabolic activity both within and between lesions at interim and EoT PET may hamper delineation quality when using the same method for all lesions and at all timepoints [15]. 

Multi-method segmentation strategies, such as majority vote (MV) and the Simultaneous Truth And Performance Level Estimate (STAPLE) probabilistic-driven framework, are promising for improving lesion delineation performance [14, 16]. The work of Berthon et al. supports this, demonstrating the successful application of a decision tree-based algorithm (ATLAAS) for tumor segmentation based on specific lesion features, a concept also validated by our group on I-PET in DLBCL using a simple SUV-based lesion-level selection rule (e.g., applying SUV4.0 for highly avid lesions and MV3 for lower-uptake lesions) [17]. The delineation performance of other more advanced statistical, machine- and deep-learning-based algorithms has mainly been tested at baseline in treatment-naive DLBCL and other lymphoma subgroups [18–26]. Hence, no single segmentation method or methodological framework has demonstrated general applicability and optimal performance across all individual DLBCL lesions, especially spanning the different treatment stages: baseline, interim, and EoT PET. The latter limitations hinder any reliable evaluation, much less the clinical implementation of (Δ)MTV as a potential response metric in DLBCL.

Therefore, this study aimed to (1) systematically evaluate how lesion- and context-related factors influence the performance of commonly used PET segmentation methods in DLBCL, and (2) develop a pragmatic lesion-adaptive Decision Rule strategy extending our previously proposed lesion-based method selection for I-PET, to select the most appropriate segmentation method for each lesion at baseline, interim and EoT PET. As a secondary benchmarking analysis, we also evaluated machine-learning classifiers as alternative selection approaches to assess whether increased model complexity provides any relevant advantage over the Decision-Rule-based strategy.

## Methods

### Patient and PET imaging selection

All newly diagnosed DLBCL patients from the multicenter randomized phase 3 HOVON-84 trial (EudraCT, 2006–005174-42, NTR10140) with both a baseline PET, a positive I-PET and a positive EoT PET scan were included, totaling 33 patients and 99 scans [27]. PET positivity was based on visual Deauville score (DS) of 4 or 5, assessed at central review by 2 independent, experienced nuclear medicine physicians and by a third reviewer if adjudication was required. Scan quality control (QC) was performed using the EANM recommendations, requiring a liver SUVmean of 1.3–3.0.3.0 and plasma glucose < 11 mmol/L. Scans were excluded from QC if lacking DICOM data or if total image activity (MBq) fell outside 50–80% of the injected FDG activity. The institutional review board approved HOVON-84 (Erasmus MC, 2007–055); all participants provided informed consent for data use in central review.

### Semi-automated segmentation methods

Six frequently used semi-automatic PET segmentation approaches were applied to all defined lymphoma lesions identified on baseline, interim and EoT PET images using the in-house developed ACCURATE (imaging analysis software) tool [28]. These methods comprised two absolute thresholds of SUVmax at 2.5 (SUV2.5) and 4.0 (SUV4.0), two relative thresholds set at 41% of SUVmax (41%max) and 50% of SUV_peak_ adapted for local background (A50peak), and two majority vote approaches segmenting voxels selected by ≥ 2 (MV2) or ≥ 3 (MV3) of the preceding 4 methods [6]. 

### Lesion selection and definition

All lymphoma lesions were included for delineation method evaluation. If lesions were closely located and appeared as a single FDG-avid cluster, the segmentation method that clustered most lesions into a single volume of interest (VOI) was used to define the lesion(cluster). SUV2.5 was excluded as a lesion(cluster) defining method due to frequent flooding, i.e., including large areas of non-lymphoma tissues. Lesions initially not selected within the defined lesion(cluster) by the remaining methods were additionally selected by the reader (PS) to those methods’ VOI.

### Segmentation performance and lesion quantification

VOIs generated by all 6 methods were independently evaluated for delineation quality by two observers (GZ, JE; both with > 5 years of experience segmenting lymphoma on PET). PET images were displayed using a 0–10 SUV intensity scale. Each method-derived delineation per lesion was scored on a 5-point quality rating scale:

- 1: No or incomplete segmentation of the lesion.
- 2: Minor underestimation of the lesion’s volume (requiring two single adjustment clicks at maximum).
- 3: Preferred segmentation of the lesion.
- 4: Minor overestimation of the lesion’s volume (requiring two single adjustment clicks at maximum).
- 5: Major overestimation of the lesion’s volume, delineation flooding into surrounding lesions/structures.

Lesion location was classified as nodal or extranodal. The nodal lesions were subdivided into lesions above and below the diaphragm. The final quality score for each delineation per lesion was based on the scores both observers agreed on. A third, experienced observer (DvA) performed final adjudication in case of discrepant observer scores.

Lesional SUVpeak, local background uptake (SUVbg), and tumor-to-background SUVpeak (TBRpeak) per lesion were based on the rate 3 method(s). SUVbg values, essential for TBRpeak calculation, were automatically determined by applying a VOI that includes the voxels located at + 1 to 1.5 cm around the rate 3 method-derived outer contour for quantification. This shell was not used to define the local background for the A50peak segmentation itself, which followed the published A50peak implementation [29]. 

### Statistical analysis

*Descriptive statistics* were used to review rating frequency distributions per method, including corresponding median uptake values and interquartile ranges (IQR), separated by imaging timepoint and location type.

*Multinomial logistic regression models* were used to study the relationship between the lesion-specific features: lesional SUVpeak, TBRpeak, SUVbg, imaging timepoint, location type, and the delineation performance of each segmentation method (with rate 3 as reference).

A *prioritizing “Decision Rule”-based approach* was explored to correctly select a single preferred (i.e., rate 3) segmentation method for each individual lesion, as previously proposed for DLBCL lesion segmentation at I-PET [14]. Over 6 million unique decision rules were systematically evaluated, each employing a pair out of the three classifying lesion features - SUVpeak, TBRpeak, and SUVbg - with specific thresholds (SUVpeak and TBRpeak from 1 to 12 in 0.5 steps, SUVbg from 0.5 to 2 in 0.2 steps). These rules applied either “>” (greater than) or “<“ (less than) prioritizing conditions and integrated three selected segmentation methods (output classes) out of the pool of 6, arranged in varied sequences. For example, a Decision Rule might state (for now arbitrary threshold values): “If lesional SUVpeak > 6.5, use Method A; if not but TBRpeak > 3, use Method B; otherwise, default to Method C”. The accuracy of the predefined Decision Rules was evaluated using 5-fold cross-validation over 50 iterations, with the dataset randomly shuffled and partitioned into 5 folds in each iteration and each fold serving once as the held-out validation fold (20%). Because the rules were predefined, no model fitting was performed. Accuracy for each predefined Decision Rule was quantified as the percentage of lesions in the held-out fold for which the rule-selected segmentation method was rated 3. The mean and standard deviation of each rule’s accuracy across the 5 folds were documented per iteration and compiled across the 50 iterations.

*As a secondary exploratory analysis*, several classical machine-learning (ML) classifiers, including Logistic Regression, Random Forest, Support Vector Machine, Decision Tree, XGBoost and Light Gradient Boosting Machine (LightGBM), were evaluated as alternative, more complex method-selection approaches to identify a preferred segmentation method per lesion, as detailed in supplementary Table 3a, b.

*Spearman’s rank correlation coefficients* were calculated to assess the relationships among MTVs (lesion-level) derived from the benchmark SUV4.0 method, the Decision Rule-based strategy, and a representative, high-performing ML-based approach relative to the reference standard, defined as the mean MTV of segmentations rated 3. Corresponding scatterplots were generated to visualize these correlations. However, as correlation metrics may fail to identify systematic volumetric discrepancies, relative and absolute differences between method-specific MTVs (lesion-level) and total MTVs (TMTV; patient-level) and corresponding reference values were calculated. Volumes were considered discrepant if the difference exceeded 10% or 3 mL, the latter consistent with the lesion threshold applied in the recent benchmark MTV study in lymphoma [13].

The analyses were performed using Rstudio (4.2.3) and Python (version 3.7.9) using scikit-learn modules.

## Results

### Descriptives of segmentation method performance

The study involved 33 patients with 99 scans and 598 lesions collectively. Of these lesions, 409 were identified at baseline, 67 at interim, and 122 at EoT PET. All lesions were segmented using the 6 semi-automatic methods, yielding 3588 scored segmentations. Observers achieved a high level of agreement in quality scores, with consistency rates ranging from 90.6% to 94.6% across segmentations per segmentation method.

**Fig. 1:**
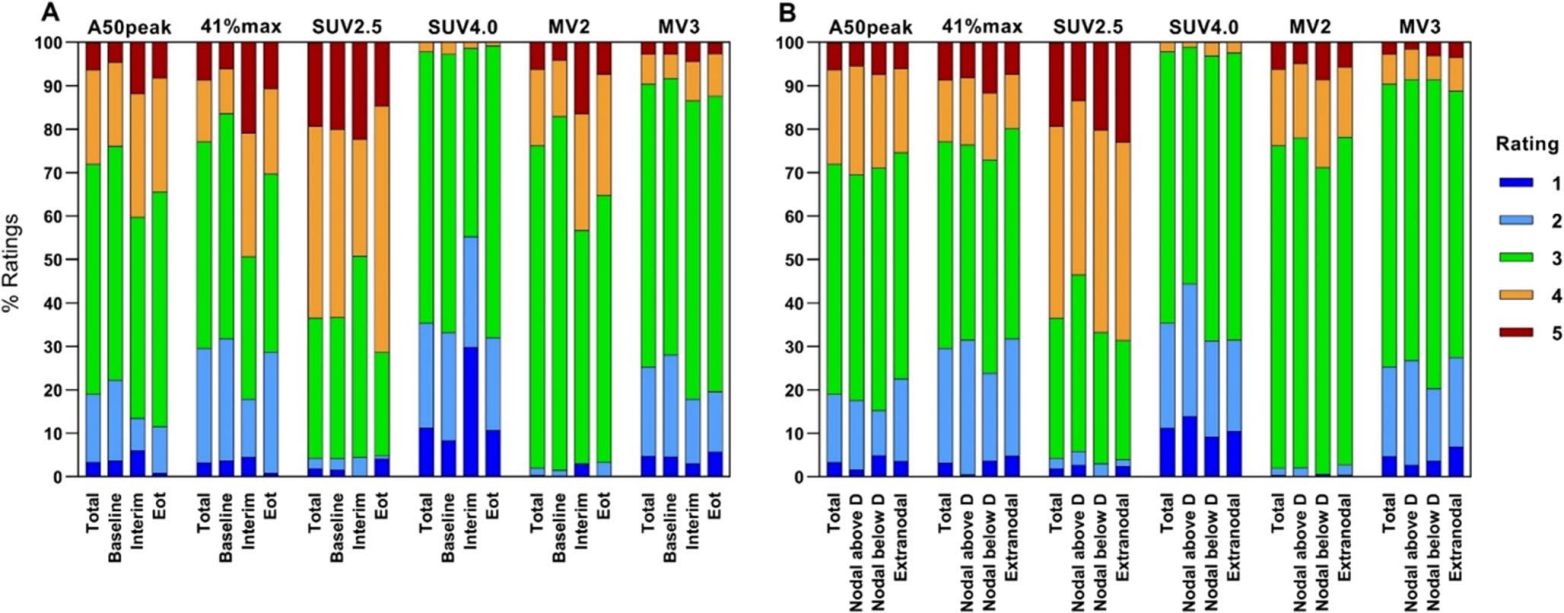
Percentages of delineation quality ratings per segmentation method displayed per imaging timepoint (**A**) and location type (**B**): nodal above diaphragm (**D**), nodal below diaphragm, and extranodal

Overall, MV2 was the best-performing segmentation method, achieving a ‘preferred segmentation’ score (rate 3) in 74.2% of lesions, followed by MV3 and SUV4.0 with success rates of 65.1% and 62.4%, respectively (Supplementary Table 1, Fig. 1). The relative threshold segmentation methods were successful in roughly half of lesions, with 53% of lesions using A50peak and 47.5% of lesions using 41%max. On the contrary, SUV2.5 proved to be the least successful, achieving a preferred delineation in only 32.3% and leading to considerable overestimation of tumor volume, or ‘flooding’ in 63.3% (ratings 4–5) of lesions.

### Descriptives of segmentation performance vs. lesional characteristics and timepoint

**Fig. 2:**
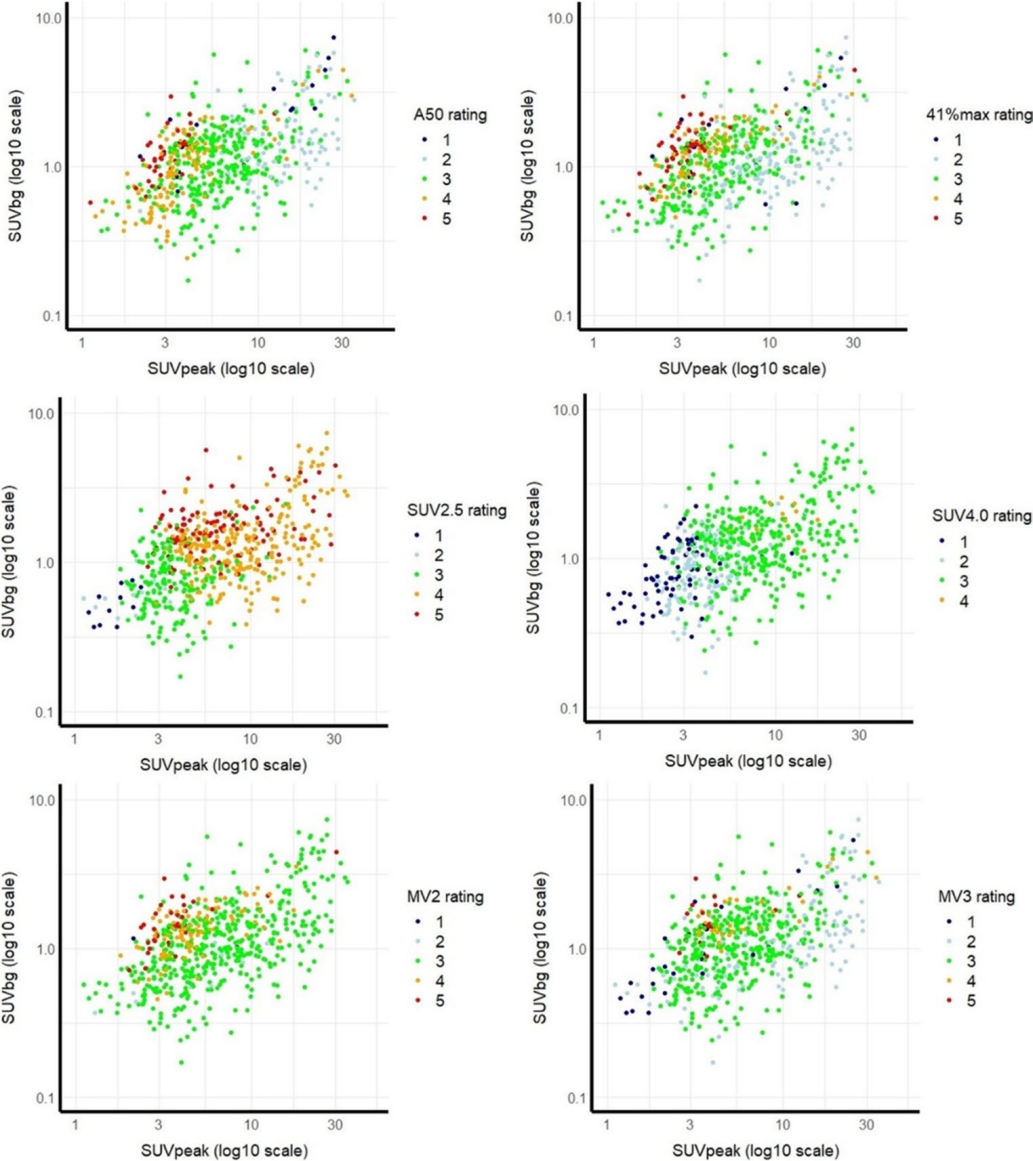
Scatterplots of SUVpeak versus SUVbackground (SUVbg) values per method and colored per rating, in which rate 3 represents a visually preferred segmentation, with rates 1–2 (colored dark and light blue) considered to be underestimating and rates 4–5 (yellow and red) overestimating of lesions’ volume

The effect of SUVpeak, TBRpeak, and SUVbg on the performance of each method exhibited distinct patterns, as displayed in Fig. 2 and Supplemental Fig. 1. The median SUVpeak, TBRpeak, and SUVbg values derived from the rate 3 segmentations varied with segmentation method, having lower values for SUV2.5 (median SUVpeak 3.4, TBRpeak 4.9, SUVbg 0.8) and higher values for SUV4.0 (7.6, 6, and 1.3). Within these ranges, the median SUV values of rate 3 segmentations derived from A50peak and 41%max were closer to those from SUV4.0. Deviations from these median values resulted in noticeable over- and under-segmentation (Supplemental Table 1, Fig. 2). The median SUV metrics of the rate 3 segmentations using MV2, potentially less frequently depending on SUV2.5 than MV3, showed slightly higher values than those from the more conservative MV3 method. The MV2 method was verified to be the most reliable, as it consistently generated rated 3 segmentations over a wide range of uptake parameters, with SUVpeak values ranging from 3.0 to 8.0 and TBRpeak values between 2.5 and 5.0.

Overall, segmentation methods performed best for lesions at baseline PET, with an average of 57.9% of segmentations rated as 3, followed by 52.6% for EoT PET and 48.5% for I-PET. At baseline, MV2 was most successful, with 81.4% of segmentations rated as 3 (Supplemental Table 1, Fig. 1). At I-PET and EoT PET, MV3 performed best, segmenting 68.7% and 68% of lesions accurately, respectively. Despite overall success, MV2 performance was most susceptible to imaging timepoint, ranging from 53.7% (I-PET) to 81.4% (baseline). In contrast, MV3 demonstrated more robustness across imaging timepoints with a mere 5.1% absolute difference (63.6% at baseline vs. 68.7% at I-PET).

### Multinomial regression of segmentation performance vs. lesional characteristics and timepoint

Several associations (*p* < 0.05) between lesional characteristics and segmentation quality performance scores across methods were observed (Supplementary Table 2). SUVpeak, TBRpeak, and less frequently SUVbg were consistently associated with segmentation performance across all methods, with varying coefficient values reflecting the differential effects of these lesional characteristics. In contrast, imaging timepoint and lesion location demonstrated less frequent and typically smaller-magnitude associations.

### Decision rule approaches

Among all tested models, simple decision rules with the following lesional features, thresholds, and prioritizing “If SUVpeak > threshold 8, use SUV4. Else, if SUVbg > 0.8, use MV3, otherwise MV2”, consistently achieved the highest mean accuracies of 0.82 (SD 0.03) across 5-fold cross-validation over 50 iterations (Supplementary Table 4).

### Machine learning classification models

In the ML analyses, models restricted to lesion-level SUVpeak, TBRpeak and SUVbg, which were identified in the multinomial regression as the main predictors of segmentation quality, achieved similar accuracies (0.79–0.82) for predicting the preferred method using a highest-probability selection strategy. Additional method-selection variants and ensemble approaches did not materially improve performance (Supplementary Table 3b).

### MTV of lesional-based methods versus preferred segmentation

**Fig. 3:**
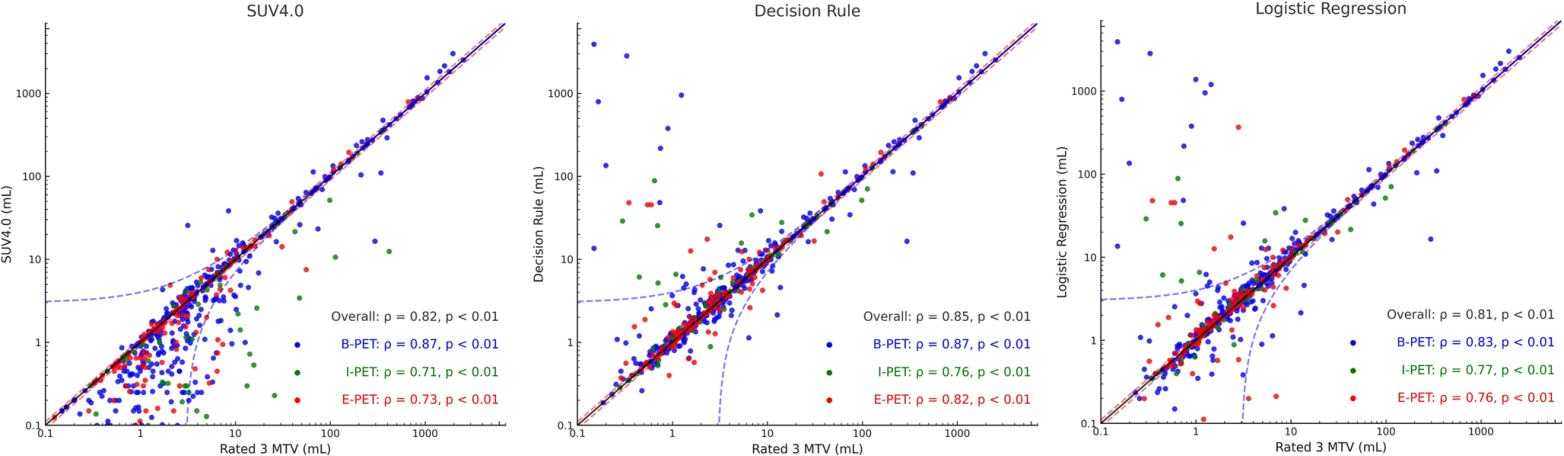
Lesion-wise scatterplots comparing MTVs obtained using SUV4.0, decision rule, and logistic regression (as the representative best-performing ML model in our analysis) with the reference MTV (average of rate 3 segmentations), plotted on log10 scales. Each point represents a lesion, with the x-axis showing the reference MTV and the y-axis the MTV from the corresponding method; data points are colored by treatment timepoint (blue: baseline PET (B-PET], green: interim PET (I-PET), red: end-of-treatment PET (E-PET)). Within each panel, Spearman correlation coefficients (ρ) and p-values are shown for the overall cohort and stratified by timepoint. The solid black line denotes the line of identity (y = x), while the dashed red and blue lines indicate relative (± 10%) and absolute (± 3 mL) deviation thresholds, respectively

Spearman’s rank correlations between lesion-wise MTVs and the reference MTV (mean volume of rate 3 segmentations) are shown in Fig. 3. Overall, the Decision Rule correlated best with the reference (ρ = 0.85), followed by SUV4.0, which served as the benchmark (ρ = 0.82), and a representative high-performing ML approach, Logistic Regression (ρ = 0.81; Supplementary Table 3c). When stratified by timepoint, correlations at baseline were very high for all three approaches, with SUV4.0 and the Decision Rule showing similar values (both ρ = 0.87) and Logistic Regression slightly lower (ρ = 0.83). At I-PET, both the Decision Rule and Logistic Regression remained strongly associated with the reference MTV (ρ = 0.76 and 0.77, respectively), compared with SUV4.0 (ρ = 0.71). At EoT PET, the Decision Rule again showed the highest correlation (ρ = 0.82), followed by Logistic Regression (ρ = 0.76) and SUV4.0 (ρ = 0.73).

Across all timepoints, SUV4.0 produced the largest number of discrepancies: 63.5% of lesions (380/598) differed by > 10% from the reference MTV and 18.1% (108/598) differed by > 3 mL. The Decision Rule substantially reduced these deviations to 46.2% of lesions with > 10% difference (276/598) and 16.4% with > 3 mL difference (98/598), whereas the Logistic Regression–based approach showed intermediate performance, with 50.2% and 16.7% of lesions outside the ± 10% and ± 3 mL limits (300/598 and 100/598), respectively. Similar patterns were seen when stratified by timepoint, with the Decision Rule consistently lowering the proportion of lesions with > 10% deviation compared with SUV4.0, particularly at interim and EoT PET.

**Fig. 4:**
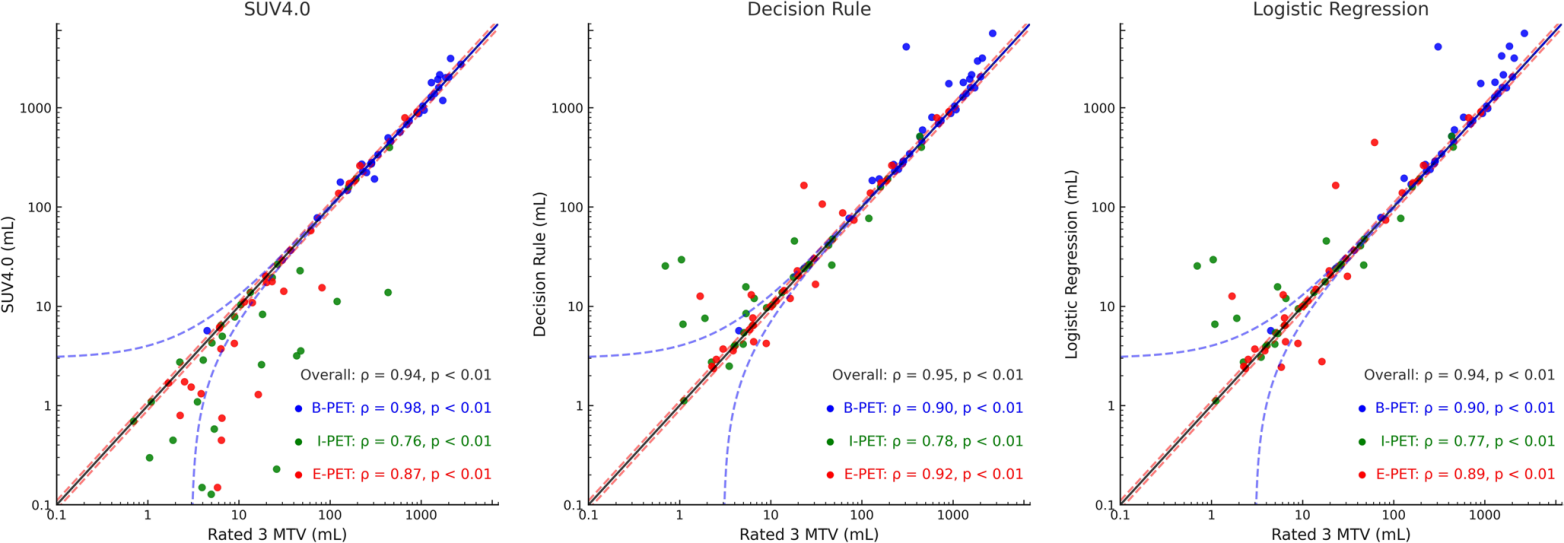
Patient-wise total metabolic tumour volume (TMTV) obtained with SUV4.0 (benchmark method), the lesion-adaptive decision rule, and a representative high-performing ML approach (logistic regression) versus the reference TMTV (mean of rate-3 segmentations), plotted on a log10 scale. Each dot represents a single PET timepoint per patient: baseline PET (B-PET, blue), interim PET (I-PET, green), and end-of-treatment PET (E-PET, red). The solid black line indicates the line of identity (y = x); the red dashed line shows the fitted regression line with 95% confidence interval (grey band). Spearman’s correlation coefficients (ρ) and p-values are shown for the overall dataset and stratified by timepoint

When evaluated at the patient level (Fig. 4), TMTVs derived with all three approaches correlated strongly with the rated-3 reference TMTVs, with overall Spearman ρ values ≥ 0.94 for the Decision Rule, SUV4.0 and Logistic Regression as high-performing representative ML approach for TMTV prediction (Supplementary Table 3c). At baseline, SUV4.0 showed the highest correlation with the reference (ρ = 0.98), whereas the Decision Rule and Logistic Regression both reached ρ = 0.90. In contrast, at I-PET the Decision Rule yielded the slightly best correlation (ρ = 0.78 vs. 0.76 for SUV4.0 and 0.77 for Logistic Regression), and a similar pattern was seen at EoT PET (ρ = 0.92 for the Decision Rule vs. 0.87 for SUV4.0 and 0.89 for Logistic Regression).

Across all timepoints, SUV4.0 showed TMTV deviations > 10% and > 3 mL relative to the reference in 58% and 60% of scans, respectively, compared with 49% and 56% for the Decision Rule and 48% and 58% for Logistic Regression. These deviations were mainly negative for SUV4.0, reflecting underestimation of TMTVs relative to the reference, whereas deviations for the Decision Rule and Logistic Regression were less frequent and more often positive, reflecting overestimation.

## Discussion

This study was designed to develop a strategy for selecting the preferred segmentation method for individual DLBCL lesions on FDG PET-based lesion-specific characteristics at different treatment timepoints. Our findings support previous research that no semi-automated delineation method is superior across all lesions and treatment timepoints [14]. Therefore, there is a need for an adaptive approach based on a model with lesion-specific input to identify the preferred segmentation method for each lesion. In this study, we addressed the latter by deriving a simple lesion-adaptive Decision Rule as the primary outcome and, only secondarily, exploring classical ML classifiers for the same task.

When considering a single segmentation method, MV2 was most consistently preferred, achieving a rate of 3 in 74.2% of segmentations overall and 81.4% at baseline assessments. SUV4.0 was previously recommended as preferred method for delineating DLBCL lesions at baseline, with performance comparable to MV2 but greater ease of use [4, 6, 9, 13]. However, in our study, SUV4.0 was considered preferred in only 64.1% of lesions. Interestingly, MV3 demonstrated better performance on I-PET, particularly for lesions with low contrast or heterogeneous metabolic activity, consistent with our previous findings on segmentation method preference for delineating DS4-5 DLBCL lesions at I-PET [14]. 

Lesion characteristics, such as SUVpeak, TBRpeak, and SUVbg, substantially affected the delineation performance score of each segmentation method, whereas imaging timepoint and lesion location type had a much smaller impact. Our findings align with Weisman, Berthon, and others, emphasizing that integrating lesional uptake metrics such as SUV and TBRpeak is crucial for improving segmentation accuracy and significantly improving the performance of machine-learning-based automated tumor delineation methods [17, 19, 30].

Using these features, a straightforward lesion-adaptive decision rule was identified that uses only two variables (SUVpeak and SUVbg) and three standard methods (SUV4.0, MV2 and MV3). This decision rule, refined from our earlier proposed strategy, replaces lesional SUVmax (> 10) with SUVpeak (> 8) as the threshold for selecting SUV4.0 and introduces a new branch for lower-uptake lesions based on SUVbg: MV3 is selected when SUVbg is above 0.8; otherwise, MV2 is preferred [14]. This approach is particularly effective because MV3, which requires agreement among three methods to include a voxel, tends to be more accurate in cases of low lesional uptake, thereby minimizing the risk of over-segmentation. However, it can lead to under-segmentation when both lesional and background uptakes are low, as both SUV4.0 and SUV2.5 may fail (or in cases of a high lesional or tumor-to-background ratio due to incomplete segmentations by A50peak and 41%max), making MV2 a better option in such scenarios due to its less strict criteria.

Despite optimization over our earlier I-PET-based strategy, the current Decision Rule still required user interaction for a small subset of lesions (18%) to ensure visually accurate delineation across all lesions. These lesions were typically small and/or low-uptake or immediately adjacent to intense physiological activity; the most extreme positive outliers in Fig. 3 arose from such cases, where the chosen threshold occasionally flooded into kidney, pericardial/myocardial or pleural regions contiguous with liver. In the rare case where repeated thresholding attempts fail to delineate a small lesion near high-physiological uptakes, a pragmatic safeguard is to use a small fixed-size VOI centred on the visually identified lesion for lesion-level or radiomics/dissemination analyses, while applying the Decision Rule to the remainder of the disease burden. Although these flooded VOIs were intentionally left uncorrected in our analysis, the decision-rule approach still yielded lesion volumes more frequently within ± 10% (and, to a lesser extent, within ± 3 mL) of the reference MTV than those obtained with the benchmark SUV4.0 method. While both methods correlated strongly with reference MTVs, SUV4.0 consistently undersegments or misses smaller lesions, more prevalent at I-PET and E-PET (Fig. 3). This tendency toward undersegmentation may necessitate more frequent manual adjustments, particularly in studies focusing on quantitative features like lesion count, DmaxBulk, and other dissemination features [31, 32].

At the patient level, TMTVs derived with SUV4.0, the Decision Rule and the best-performing ML approach all showed firm overall agreement with the reference, indicating that lesion-level biases partly, but not completely, cancel when volumes are summed. However, at interim and EoT PET the Decision Rule provided slightly better TMTV agreement and fewer negative deviations (underestimation) than SUV4.0, which is notable given that the limited number of substantial outliers of the Decision Rule were not manually corrected, in contrast to typical clinical workflows where such cases would be adjusted.

In this multi-class setting, using classical ML classifiers for automated method selection is challenging, because several segmentation methods can be acceptable (rate 3) for the same lesion and there is no single uniquely correct class for model training. Even so, all classical ML classifiers we evaluated achieved similar accuracies (0.79–0.82) with a highest-probability strategy for preferred method selection, and more complex, elaborate schemes (hierarchical rules, restricted method sets, stacked ensembles) did not materially improve performance. Overall, these findings indicate that, in our current setting, increasing algorithmic complexity does not necessarily translate into better method selection than the pragmatic lesion-adaptive Decision Rule.

At the same time, the limited gain from more complex models might also reflect the restricted set of input variables we used. To maintain simplicity and implementability, we confined our models to practical, routinely available inputs with the location variable contributing little beyond the uptake metrics, as expected for a non-organ-aware metric. Likewise, SUVbg is derived from a generic local background shell and, although it captures local background activity to some extent, cannot fully represent organ-specific uptake patterns. More advanced lesion-level features (e.g., shape, texture, intralesional heterogeneity), combined with explicit anatomical context, could reasonably be expected to improve preferred-method prediction by better capturing both lesion characteristics and organ-dependent background activity patterns, thereby more closely approximating the anatomical/contextual reasoning implicit in expert visual ratings [17]. Therefore, future work could include such extensions on top of the current lesion-adaptive presegmentation framework, once larger, well-annotated lymphoma datasets across treatment phases are available.

It is important to acknowledge that various other advanced, machine- or deep learning-based semi- and full-automated lesion segmentation algorithms have been proposed in DLBCL and other FDG-avid lymphomas [19, 30]. While several AI-based models for TMTV predictions show considerable promise, most have been trained and validated on baseline scans from single-centre cohorts, with single-observer “ground truth” and presegmentation-based VOIs derived from a single method, with limited assessment across treatment phases or observers. Their generalisability to DS4-5 DLBCL at interim and EoT PET is therefore limited. In HOVON-84, only 33 of 574 patients had DS4-5-positive scans at both these timepoints. Still, these yielded 598 lesions and 3,588 rated segmentations, which we consider adequate for the technical question addressed, while recognising that external validation of the Decision Rule on technical generalisability and reproducibility in larger, independent cohorts will be essential [27]. 

At baseline, SUV4.0 and the Decision Rule produced highly concordant TMTVs. Substituting the Decision Rule for SUV4.0 is thus unlikely to change established baseline models such as the IMPI materially. At present, however, there are no validated prognostic or treatment‑predictive models in DLBCL that incorporate interim/EoT TMTV or longitudinal TMTV change, so we cannot yet determine whether the Decision Rule improves prognostic performance over SUV4.0 at these timepoints and whether this will be affected by missing or underestimating smaller lesions when using SUV4.0.

Developing prognostic and predictive PET models for (early) treatment-adaptive strategies that explicitly incorporate interim and EoT PET-based MTV or other radiomics requires large, high-quality datasets, particularly because increased FDG uptake at these timepoints may partly reflect inflammation/repair, making MTV/TMTV and derived radiomics biologically more complex and their prognostic value potentially harder to establish even with expert-guided segmentations. However, this subgroup of incomplete DLBCL responders, characterized by significantly poorer outcomes, is rare. Consequently, adequately powered prognostic analyses will require pooling of multiple small patient cohorts across centres and trials, which is only scientifically meaningful if lesion delineation methodology is harmonised so that MTV/TMTV and dissemination PET metrics are comparable. Early delta‑radiomics studies in DS4-5-positive subsets reported promising signals, especially for dissemination features, but all identified non-standardised lesion delineation beyond baseline as a major limitation [33–36]. Notably, two such studies applied a 41% SUVmax threshold at I-PET, an approach that performed poorly at these timepoints in our data (Supplementary Table 1). Moreover, Baseline MIP-CNN work on HOVON-84, externally validated on PETAL and extended across additional trials, showed that a segmentation-free deep learning model was outperformed by segmentation-dependent PET and clinical PET models incorporating MTV, DmaxBulk, and SUVpeak, in predicting 2-year time-to-progression [37]. This explicitly supports the relevance of DLBCL segmentations for improving prognostically valuable radiomics-ML and DL models at baseline PET and further underscores the need for rigorous segmentation methodology when extending such models to interim and EoT PET.

The present lesion-adaptive Decision Rule can be viewed as a complementary, transparent tool that improves and standardises the semi-automatic selection of the preferred segmentation method for individual DLBCL lesions, regardless of treatment phase, and may serve as a predelineation tool for radiomics-ML or DL-based PET model development in future prognostic or response-adapted DLBCL studies. Reducing method-related variability in lesion segmentation, particularly at interim and EoT PET, helps to bridge the current methodological gap between baseline-focused segmentation practice and the needs of longitudinal, treatment-adaptive PET studies in DLBCL.

## Conclusions

A decision rule approach using lesional SUVpeak and background SUV as inputs achieved a high performance score in identifying a preferred segmentation method for individual DLBCL lesions, regardless of treatment phase. This straightforward, semi-automatic strategy performs as well as, or even better than, more complex machine learning models, and improves on SUV4.0, particularly for interim and end-of-treatment PET. Although supervision remains necessary, its simplicity and transparency make it easy to use and widely applicable, helping to close the current gap in segmentation methodology beyond baseline PET and providing a pragmatic basis for improving future machine- and deep-learning PET studies in DLBCL.

## Supplementary Information

Below is the link to the electronic supplementary material.

Supplementary figure 1 (PNG 1.03 MB)

High Resolution Image (TIF 1.04 MB)

Supplementary figure 2 (PNG 1.37 MB)

High Resolution Image (TIF 774 KB)

ESM 3 (DOCX 24.7 KB)

ESM 4 (DOCX 37.1 KB)

ESM 5 (DOCX 25.9 KB)

ESM 6 (DOCX 16.3 KB)

## Data Availability

The PET/CT imaging datasets (DICOM) and per-lesion segmentations (NIfTI) generated and analyzed during the current study are available from the corresponding author on reasonable request.

## References

[1] Alduaij W, Collinge B, Ben-Neriah S, Jiang A, Hilton LK, Boyle M et al. Molecular determinants of clinical outcomes in a real-world diffuse large B-cell lymphoma population. Blood [Internet]. 2023; 141(20):2493–507. Available from: 10.1182/blood.2022018248 10.1182/blood.2022018248 36302166

[2] Eertink JJ, Arens AIJ, Huijbregts JE, Celik F, de Keizer B, Stroobants S et al. Aberrant patterns of PET response during treatment for DLBCL patients with MYC gene rearrangements. Eur J Nucl Med Mol Imaging [Internet]. 2022; 49(3):943–52. Available from: https://pubmed.ncbi.nlm.nih.gov/34476551/10.1007/s00259-021-05498-7 PMC8803795 34476551

[3] Ilyas H, Mikhaeel NG, Dunn JT, Rahman F, Møller H, Smith D, et al. Defining the optimal method for measuring baseline metabolic tumour volume in diffuse large B-cell lymphoma. Eur J Nucl Med Mol Imaging. 2018;45(7):1142–54. 29460024 10.1007/s00259-018-3953-z PMC5953976

[4] Burggraaff CN, Rahman F, Kaßner I, Pieplenbosch S, Barrington SF, Jauw YWS, et al. Optimizing workflows for fast and reliable metabolic tumor volume measurements in diffuse large B cell lymphoma. Mol Imaging Biol. 2020;22(4):1102–10. 31993925 10.1007/s11307-020-01474-z PMC7343740

[5] Guo B, Tan X, Ke Q, Cen H. Prognostic value of baseline metabolic tumor volume and total lesion glycolysis in patients with lymphoma: a meta-analysis. PLoS One. 2019;14(1): e0210224. 10.1371/journal.pone.0210224. 10.1371/journal.pone.0210224 PMC6326501 30625203

[6] Barrington S, Zwezerijnen BG, de Vet HC, Heymans M, Mikhaeel NG, Burggraaff C, et al. Automated segmentation of baseline metabolic total tumor burden in diffuse large B-cell lymphoma: which method is most successful? J Nucl Med. 2021;62(3):332–7. 10.2967/jnumed.119.238923 10.2967/jnumed.119.238923 PMC8049348 32680929

[7] Malek E, Sendilnathan A, Yellu M, Petersen A, Fernandez-Ulloa M, Driscoll JJ. Metabolic tumor volume on interim PET is a better predictor of outcome in diffuse large B-cell lymphoma than semiquantitative methods. Blood Cancer J. 2015;5(7):e326. 10.1038/bcj.2015.51. 10.1038/bcj.2015.51 PMC4526777 26207787

[8] Richter J, Hüttmann A, Rekowski J, Schmitz C, Gärtner S, Ose C, et al. Molecular characteristics of diffuse large B-cell lymphoma and correlation with baseline metabolic tumor volume (MTV), interim positron emission tomography (iPET) and outcome in the PETAL trial. Blood. 2018;132(Supplement 1):4188–4188.

[9] Mikhaeel NG, Heymans MW, Eertink JJ, de Vet HCW, Boellaard R, Dührsen U, et al. Proposed new dynamic prognostic index for diffuse large B-cell lymphoma: international metabolic prognostic index. J Clin Oncol. 2022;40(21):2352–60. 10.1200/jco.21.02063. 35357901 10.1200/JCO.21.02063 PMC9287279

[10] Islam P, Goldstein J, Flowers CR. PET-derived tumor metrics predict DLBCL response and progression-free survival. Leuk Lymphoma. 2019;60(8):1965–71. 30714446 10.1080/10428194.2018.1562181 PMC6635064

[11] Wang H, Shen G, Jiang C, Li L, Cui F, Tian R. Prognostic value of baseline, interim and end-of-treatment18F-FDG PET/CT parameters in extranodal natural killer/T-cell lymphoma: a meta-analysis. PLoS ONE. 2018;13(3):1–14. 10.1371/journal.pone.0194435 PMC5860776 29558489

[12] Baratto L, Wu F, Minamimoto R, Hatami N, Liang T, Sabile J et al. Correlation of 18-fluorodeoxyglucose PET/computed tomography parameters and clinical features to predict outcome for diffuse large B-cell lymphoma. Nucl Med Commun [Internet]. 2021;42(7):792–9. Available from: https://pubmed.ncbi.nlm.nih.gov/33741852/10.1097/MNM.0000000000001398 33741852

[13] Boellaard R, Buvat I, Ceriani L, Cottereau AS, Guerra L, Hicks R et al. International benchmark for total metabolic tumor volume assessment in baseline FDG PET/CT of lymphoma patients. Journal of Nuclear Medicine. 2023;64(supplement 1). 10.2967/jnumed.124.267789 PMC11372260 39089812

[14] Zwezerijnen GJC, Eertink JJ, Burggraaff CN, Wiegers SE, Shaban EA, Pieplenbosch S, et al. Interobserver agreement on automated metabolic tumor volume measurements of Deauville score 4 and 5 lesions at interim ^18^F-FDG PET in diffuse large B-cell lymphoma. J Nucl Med. 2021;62(11):1531–6. 10.2967/jnumed.120.258673. 33674403 10.2967/jnumed.120.258673 PMC8612315

[15] Cheebsumon P, Yaqub M, Van Velden FHP, Hoekstra OS, Lammertsma AA, Boellaard R. Impact of [18F]FDG PET imaging parameters on automatic tumour delineation: need for improved tumour delineation methodology. Eur J Nucl Med Mol Imaging [Internet]. 2011;38(12):2136. Available from: http://pmc/articles/PMC3228515/. 10.1007/s00259-011-1899-5 PMC3228515 21858528

[16] Dewalle-Vignion AS, Betrouni N, Baillet C, Vermandel M. Is STAPLE algorithm confident to assess segmentation methods in PET imaging? Phys Med Biol. 2015;60(24):9473–91. 26584044 10.1088/0031-9155/60/24/9473

[17] Berthon B, Marshall C, Evans M, Spezi E. ATLAAS: an automatic decision tree-based learning algorithm for advanced image segmentation in positron emission tomography. Phys Med Biol. 2016;61(13):4855–69. 27273293 10.1088/0031-9155/61/13/4855

[18] Capobianco N, Meignan M, Cottereau A, Vercellino L, Sibille L, Spottiswoode B, et al. Deep-learning ^18^F-FDG uptake classification enables total metabolic tumor volume estimation in diffuse large B-cell lymphoma. J Nucl Med. 2020;62(1):30. 10.2967/jnumed.120.242412. 32532925 10.2967/jnumed.120.242412 PMC8679589

[19] Weisman AJ, Kieler M, Perlman S, Hutchings M, Jeraj R, Kostakoğlu L et al. Comparison of 11 automated PET segmentation methods in lymphoma. Phys Med Biol [Internet]. 2020;65(23):235019. Available from: 10.1088/1361-6560/abb6bd 10.1088/1361-6560/abb6bd 32906088

[20] Constantino CS, Leocádio S, Oliveira FPM, Silva M, Oliveira C, Castanheira J, et al. Evaluation of semiautomatic and deep learning–based fully automatic segmentation methods on [18F]FDG PET/CT images from patients with lymphoma: influence on tumor characterization. J Digit Imaging. 2023;36(4):1864–76. 10.1007/s10278-023-00823-y. 37059891 10.1007/s10278-023-00823-y PMC10407010

[21] Yousefirizi F, Klyuzhin IS, O JH, Harsini S, Tie X, Shiri I et al. TMTV-Net: fully automated total metabolic tumor volume segmentation in lymphoma PET/CT images - a multi-center generalizability analysis. Eur J Nucl Med Mol Imaging [Internet]. Available from: http://www.ncbi.nlm.nih.gov/pubmed/38326655 10.1007/s00259-024-06616-x 38326655

[22] Jiang C, Chen K, Teng Y, Ding C, Zhou Z, Gao Y, et al. Deep learning–based tumour segmentation and total metabolic tumour volume prediction in the prognosis of diffuse large B-cell lymphoma patients in 3D FDG-PET images. Eur Radiol. 2022;32(7):4801–12. 35166895 10.1007/s00330-022-08573-1

[23] Revailler W, Cottereau AS, Rossi C, Noyelle R, Trouillard T, Morschhauser F et al. Deep learning approach to automatize TMTV calculations regardless of segmentation methodology for major FDG-avid lymphomas. Diagnostics (Basel). 2022;12(2). Available from: https://pubmed.ncbi.nlm.nih.gov/35204515/10.3390/diagnostics12020417 PMC8870809 35204515

[24] Girum KB, Rebaud L, Cottereau AS, Meignan M, Clerc J, Vercellino L et al. 18F-FDG PET maximum-intensity projections and artificial intelligence: a win-win combination to easily measure prognostic biomarkers in DLBCL patients. J Nucl Med [Internet]. 2022;63(12):1925–32. Available from: https://pubmed.ncbi.nlm.nih.gov/35710733/10.2967/jnumed.121.263501 PMC9730929 35710733

[25] Jemaa S, Paulson JN, Hutchings M, Kostakoglu L, Trotman J, Tracy S et al. Full automation of total metabolic tumor volume from FDG-PET/CT in DLBCL for baseline risk assessments. Cancer Imaging [Internet]. 2022;22(1). Available from: https://pubmed.ncbi.nlm.nih.gov/35962459/10.1186/s40644-022-00476-0 PMC9373298 35962459

[26] Blanc-Durand P, Jégou S, Kanoun S, Berriolo-Riedinger A, Bodet-Milin C, Kraeber-Bodéré F, et al. Fully automatic segmentation of diffuse large B cell lymphoma lesions on 3D FDG-PET/CT for total metabolic tumour volume prediction using a convolutional neural network. Eur J Nucl Med Mol Imaging. 2021;48(5):1362–70. 33097974 10.1007/s00259-020-05080-7

[27] Lugtenburg PJ, de Nully Brown P, van der Holt B, et al. Rituximab-CHOP with early rituximab intensification for diffuse large B-cell lymphoma: a randomized phase III trial of the HOVON and the nordic lymphoma group (HOVON-84). J Clin Oncol. 2020;38(29):3377–87. 10.1200/JCO.19.03418. 10.1200/JCO.19.03418 32730183

[28] Boellaard R. Quantitative oncology molecular analysis suite: ACCURATE. J Nucl Med [Internet]. 2018;59(supplement 1):1753. Available from: https://researchinformation.amsterdamumc.org/en/publications/quantitative-oncology-molecular-analysis-suite-accurate

[29] Cheebsumon P, Yaqub M, Van Velden FHP, Hoekstra OS, Lammertsma AA, Boellaard R. Impact of [18F]FDG PET imaging parameters on automatic tumour delineation: Need for improved tumour delineation methodology. Eur J Nucl Med Mol Imaging [Internet]. 2011;38(12):2136–44. Available from: https://pubmed.ncbi.nlm.nih.gov/21858528/10.1007/s00259-011-1899-5 PMC3228515 21858528

[30] Veziroglu EM, Farhadi F, Hasani N, Nikpanah M, Roschewski M, Summers RM, et al. Role of artificial intelligence in PET/CT imaging for management of lymphoma. Semin Nucl Med. 2023;53(3):426–48. 10.1053/j.semnuclmed.2022.11.003. 36870800 10.1053/j.semnuclmed.2022.11.003

[31] Cottereau A, Meignan M, Nioche C, Capobianco N, Clerc J, Chartier L, et al. Risk stratification in diffuse large B-cell lymphoma using lesion dissemination and metabolic tumor burden calculated from baseline PET/CT†. Ann Oncol. 2021;32(3):404–11. 10.1016/j.annonc.2020.11.019. 33278600 10.1016/j.annonc.2020.11.019

[32] Eertink JJ, Zwezerijnen GJC, Heymans MW, Pieplenbosch S, Wiegers SE, Dührsen U et al. Baseline PET radiomics outperforms the IPI risk score for prediction of outcome in diffuse large B-cell lymphoma. Blood [Internet]. 2023;141(25):3055–64. Available from: https://pubmed.ncbi.nlm.nih.gov/37001036/10.1182/blood.2022018558 PMC10646814 37001036

[33] Dang J, Peng X, Wu P, Yao Y, Tan X, Ye Z et al. Predictive value of Dmax and %∆SUVmax of 18F-FDG PET/CT for the prognosis of patients with diffuse large B-cell lymphoma. BMC Med Imaging [Internet]. 2023;23(1):1–9. Available from: https://bmcmedimaging.biomedcentral.com/articles/10.1186/s12880-023-01138-8 10.1186/s12880-023-01138-8 PMC10617085 37907837

[34] Cui Y, Li Y, Hu W, Wu Z, Li S, Wang H. Evaluating ∆MTV%, ∆Dmax%, and %∆SUVmax of 18F-FDG PET/CT for mid-treatment efficacy and prognosis in diffuse large B-cell lymphoma. Discov Oncol [Internet]. 2025;16(1):1–10. Available from: https://link.springer.com/10.1007/s12672-025-02126-w 10.1007/s12672-025-02126-w PMC11950622 40146454

[35] Cui Y, Jiang Y, Deng X, Long W, Liu B, Fan W et al. 18F-FDG PET-based combined baseline and end-of-treatment radiomics model improves the prognosis prediction in diffuse large b cell lymphoma after first-line therapy. Acad Radiol [Internet]. 2023;30(7):1408–18. Available from: https://www.sciencedirect.com/science/article/abs/pii/S1076633222005487 10.1016/j.acra.2022.10.011 36437191

[36] Mitura J, Jóźwiak R, Chrapko B, Bachanek-Mitura O, Wybrańska J, PET/CT. Automated evaluation of lymphoma treatment response: integrating delta radiomics and deep learning from 2-[18F]FDG. 2025. Available from: https://papers.ssrn.com/abstract=5062563

[37] Ferrández MC, Golla SSV, Eertink JJ, et al. Validation of an artificial intelligence-based prediction model using 5 external PET/CT datasets of diffuse large B-cell lymphoma. J Nucl Med. 2024;65(11):1802–7. 10.2967/jnumed.124.268191. 39362767 10.2967/jnumed.124.268191

