# Supplementary figures and images for: A Lesion-adaptive Segmentation Approach for Tumor Delineation on FDG PET/CT in Diffuse Large B-cell Lymphoma Patients
Source: Eur J Nucl Med Mol Imaging. 2026 Feb 14;53(6):4175–85. doi: 10.1007/s00259-026-07768-8 (PMC13121395; doi:10.1007/s00259-026-07768-8)

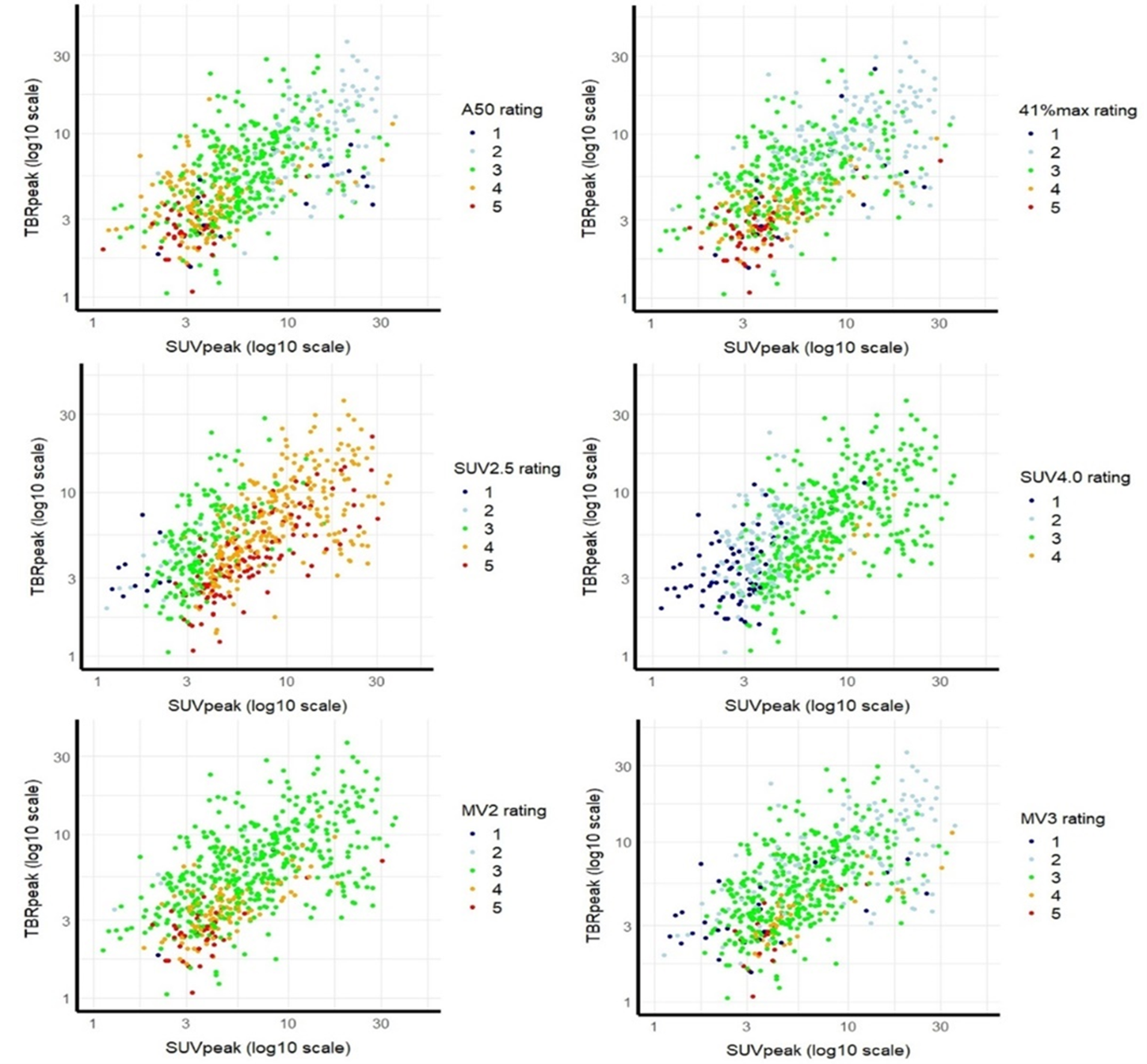

Supplement: Supplementary file 1 — (PNG 1.03 MB) [file 259_2026_7768_Fig5_ESM.png]

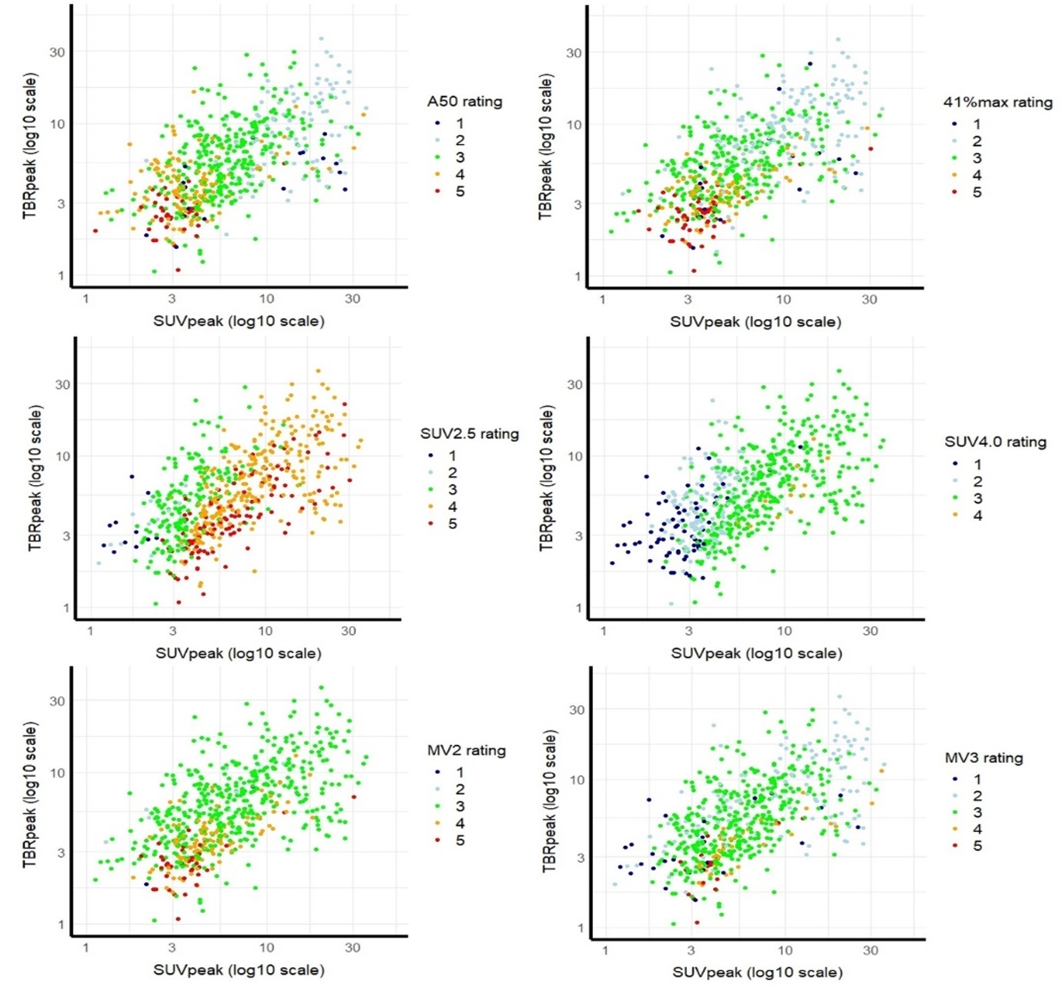

Supplement: Supplementary file 2 — High Resolution Image (TIF 1.04 MB) [file 259_2026_7768_MOESM1_ESM.tif]

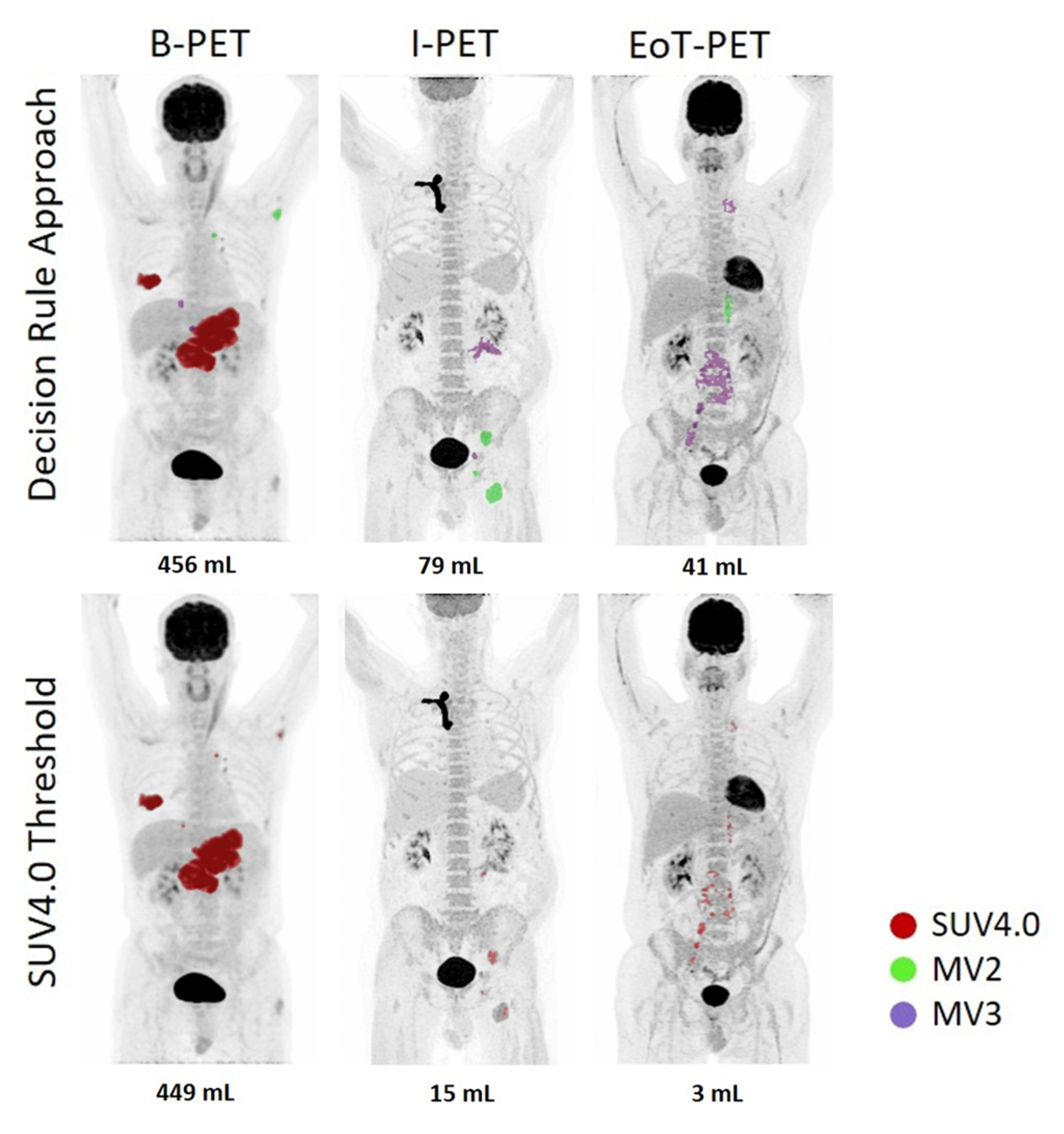

Supplement: Supplementary file 3 — (PNG 1.37 MB) [file 259_2026_7768_Fig6_ESM.png]

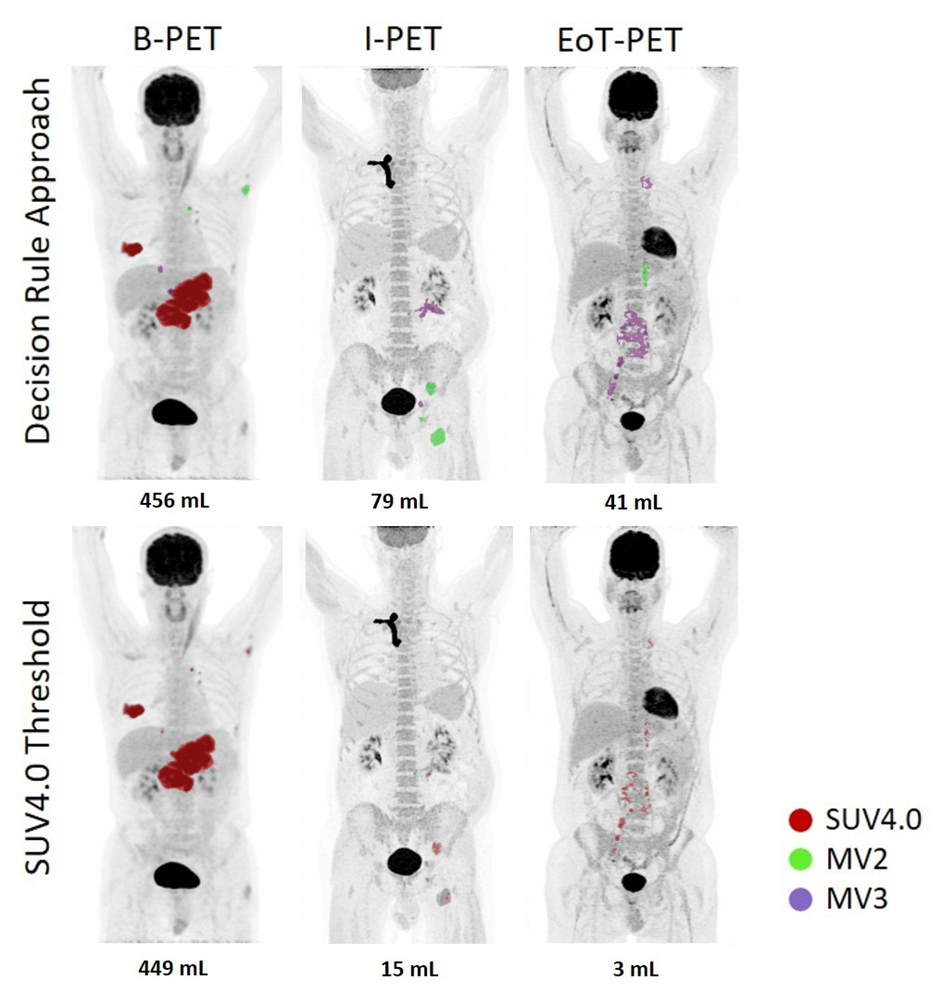

Supplement: Supplementary file 4 — High Resolution Image (TIF 774 KB) [file 259_2026_7768_MOESM2_ESM.tif]
