# Supplementary material for: A Lesion-adaptive Segmentation Approach for Tumor Delineation on FDG PET/CT in Diffuse Large B-cell Lymphoma Patients
Source: Eur J Nucl Med Mol Imaging. 2026 Feb 14;53(6):4175–85. doi: 10.1007/s00259-026-07768-8 (PMC13121395; doi:10.1007/s00259-026-07768-8)
Supplement: Supplementary file 5 — (DOCX 24.7 KB) [file 259_2026_7768_MOESM3_ESM.docx]

**Supplemental Table 1.** Median and interquartile ranges of segmentation rates for each method, separated by timepoint and location type. TBRpeak; TumorSUVpeak-to-backgroundSUVpeak, SUVbg; backgroundSUVpeak, IQR; interquartile range, D; diaphragm, * percentage of rating numbers per total number of ratings per timepoint, ** percentage of rating numbers per total number of ratings per location.

|  |  |  | **SUVpeak median (IQR)** | **TBRpeak median (IQR)** | **SUVbg median (IQR)** | **Timing N (%*)** | | | **Location N (%**)** | | |
| --- | --- | --- | --- | --- | --- | --- | --- | --- | --- | --- | --- |
| **Method** | **Rating** | **N (%)** |  |  |  | **Baseline** | **Interim** | **EoT** | **Nodal above D** | **Nodal below D** | **Extranodal** |
| **A50peak** | 1 | 20 (3.3) | 4.3 (13.4) | 3.8 (2.9) | 1.7 (1.5) | 15 (3.7) | 4 (6) | 1 (0.8) | 3 (1.6) | 8 (4.9) | 9 (3.6) |
|  | 2 | 94 (15.7) | 14.2 (10.4) | 9.5 (8.9) | 1.4 (1) | 76 (18.6) | 5 (7.5) | 13 (10.7) | 30 (16) | 17 (10.4) | 47 (19) |
|  | **3** | **317 (53)** | **5.7 (4.1)** | **5.7 (4.6)** | **1 (0.8)** | **220 (53.8)** | **31 (46.3)** | **66 (54.1)** | **97 (51.9)** | **91 (55.8)** | **129 (52)** |
|  | 4 | 130 (21.7) | 3.4 (1.7) | 4 (2.1) | 1 (0.7) | 79 (19.3) | 19 (28.4) | 32 (26.2) | 47 (25.1) | 35 (21.5) | 48 (19.4) |
|  | 5 | 37 (6.2) | 2.9 (0.9) | 2.5 (1) | 1.1 (0.5) | 19 (4.6) | 8 (11.9) | 10 (8.2) | 10 (5.3) | 12 (7.4) | 15 (6) |
| **41%max** | 1 | 19 (3.2) | 4 (8.3) | 3.8 (3.4) | 1.3 (1) | 15 (3.7) | 3 (4.5) | 1 (0.8) | 1 (0.5) | 6 (3.7) | 12 (4.8) |
|  | 2 | 158 (26.4) | 10.1 (11.7) | 8.4 (7.2) | 1.1 (0.9) | 115 (28.1) | 9 (13.4) | 34 (27.9) | 58 (31) | 33 (20.2) | 67 (27) |
|  | **3** | **284 (47.5)** | **5.2 (4.6)** | **5.3 (3.9)** | **1 (0.8)** | **212 (51.8)** | **22 (32.8)** | **50 (41)** | **84 (44.9)** | **80 (49.1)** | **120 (48.4)** |
|  | 4 | 85 (14.2) | 4.5 (2.3) | 3.6 (1.4) | 1.3 (0.7) | 42 (10.3) | 19 (28.4) | 24 (19.7) | 29 (15.5) | 25 (15.3) | 31 (12.5) |
|  | 5 | 52 (8.7) | 3.4 (1.1) | 2.6 (0.7) | 1.4 (0.6) | 25 (6.1) | 14 (20.9) | 13 (10.7) | 15 (8) | 19 (11.7) | 18 (7.3) |
| **SUV2.5** | 1 | 11 (1.8) | 1.7 (0.6) | 2.9 (0.9) | 0.5 (0.2) | 6 (1.5) | NA | 5 (4.1) | 5 (2.7) | NA | 6 (2.4) |
|  | 2 | 15 (2.5) | 2.4 (1.4) | 3.5 (1.7) | 0.6 (0.4) | 11 (2.7) | 3 (4.5) | 1 (0.8) | 6 (3.2) | 5 (3.1) | 4 (1.6) |
|  | **3** | **193 (32.3)** | **3.4 (1.6)** | **4.9 (4.2)** | **0.8 (0.5)** | **133 (32.5)** | **31 (46.3)** | **29 (23.8)** | **76 (40.6)** | **49 (30.1)** | **68 (27.4)** |
|  | 4 | 264 (44.1) | 8.5 (8.8) | 6.7 (6.2) | 1.3 (0.7) | 177 (43.3) | 18 (26.9) | 69 (56.6) | 75 (40.1) | 76 (46.6) | 113 (45.6) |
|  | 5 | 115 (19.2) | 6.4 (6.6) | 3.9 (3.4) | 1.6 (0.7) | 82 (20) | 15 (22.4) | 18 (14.8) | 25 (13.4) | 33 (20.2) | 57 (23) |
| **SUV4.0** | 1 | 67 (11.2) | 2.6 (1.2) | 3.2 (2) | 0.8 (0.5) | 34 (8.3) | 20 (29.9) | 13 (10.7) | 26 (13.9) | 15 (9.2) | 26 (10.5) |
|  | 2 | 145 (24.2) | 3.7 (1.5) | 4.7 (3.3) | 0.8 (0.5) | 102 (24.9) | 17 (25.4) | 26 (21.3) | 57 (30.5) | 36 (22.1) | 52 (21) |
|  | **3** | **373 (62.4)** | **7.6 (8.1)** | **6 (6)** | **1.3 (0.8)** | **262 (64.1)** | **29 (43.3)** | **82 (67.2)** | **102 (54.5)** | **107 (65.6)** | **164 (66.1)** |
|  | 4 | 13 (2.2) | 10.9 (4.9) | 5.5 (2.1) | 1.8 (0.5) | 11 (2.7) | 1 (1.5) | 1 (0.8) | 2 (1.1) | 5 (3.1) | 6 (2.4) |
|  | 5 | NA | NA | NA | NA | NA | NA | NA | NA | NA | NA |
| **MV2** | 1 | 2 (0.3) | 5.2 (3.1) | 5 (3.1) | 1.1 (0.1) | NA | 2 (3) | NA | NA | 1 (0.6) | 1 (0.4) |
|  | 2 | 10 (1.7) | 6.1 (3.9) | 7 (4.4) | 0.7 (0.5) | 6 (1.5) | NA | 4 (3.3) | 4 (2.1) | NA | 6 (2.4) |
|  | **3** | **444 (74.2)** | **6.2 (7.1)** | **6 (5.2)** | **1 (0.8)** | **333 (81.4)** | **36 (53.7)** | **75 (61.5)** | **142 (75.9)** | **115 (70.6)** | **187 (75.4)** |
|  | 4 | 105 (17.6) | 4.5 (2.2) | 3.6 (1.6) | 1.3 (0.6) | 53 (13) | 18 (26.9) | 34 (27.9) | 32 (17.1) | 33 (20.2) | 40 (16.1) |
|  | 5 | 37 (6.2) | 3.5 (1.3) | 2.5 (0.8) | 1.4 (0.6) | 17 (4.2) | 11 (16.4) | 9 (7.4) | 9 (4.8) | 14 (8.6) | 14 (5.6) |
| **MV3** | 1 | 28 (4.7) | 3.3 (2.3) | 3.1 (2.3) | 0.9 (0.8) | 19 (4.6) | 2 (3) | 7 (5.7) | 5 (2.7) | 6 (3.7) | 17 (6.9) |
|  | 2 | 123 (20.6) | 10.8 (15.2) | 7.8 (8.6) | 1.1 (0.9) | 96 (23.5) | 10 (14.9) | 17 (13.9) | 45 (24.1) | 27 (16.6) | 51 (20.6) |
|  | **3** | **389 (65.1)** | **5.4 (4.6)** | **5.1 (4.5)** | **1.1 (0.7)** | **260 (63.6)** | **46 (68.7)** | **83 (68)** | **121 (64.7)** | **116 (71.2)** | **152 (61.3)** |
|  | 4 | 41 (6.9) | 4.7 (2) | 4 (2.1) | 1.4 (0.5) | 23 (5.6) | 6 (9) | 12 (9.8) | 13 (7) | 9 (5.5) | 19 (7.7) |
|  | 5 | 17 (2.8) | 3.7 (0.9) | 2.6 (1.1) | 1.5 (0.7) | 11 (2.7) | 3 (4.5) | 3 (2.5) | 3 (1.6) | 5 (3.1) | 9 (3.6) |
