# Supplementary material for: A Lesion-adaptive Segmentation Approach for Tumor Delineation on FDG PET/CT in Diffuse Large B-cell Lymphoma Patients
Source: Eur J Nucl Med Mol Imaging. 2026 Feb 14;53(6):4175–85. doi: 10.1007/s00259-026-07768-8 (PMC13121395; doi:10.1007/s00259-026-07768-8)
Supplement: Supplementary file 6 — (DOCX 37.1 KB) [file 259_2026_7768_MOESM4_ESM.docx]

**Supplemental Table 2.** Multinomial logistic regression coefficients (β) for lesion-level variables SUVpeak, TBRpeak, background SUV (SUVbg), imaging time point (baseline PET [reference], interim PET, end-of-treatment [EoT] PET) and lesion location (nodal above diaphragm [reference], nodal below diaphragm, extranodal) on segmentation ratings 1, 2, 4 and 5, with rating 3 as the reference category. For each non-reference rating, the coefficients show how the log of the probability ratio P(rating = k)/P(rating = 3) changes per one-unit increase in the corresponding predictor (for continuous predictors) or relative to the reference category (for categorical predictors), assuming all other predictors remain unchanged. Assumption testing before multinomial logistic regression analyses demonstrated an absence of collinearity among the lesional characteristic variables, including time points. The variance inflation factors (VIFs) for the remaining variables were within acceptable limits: SUVpeak (VIF = 4.70), TBRpeak (VIF = 3.16), SUVbg (VIF = 3.75), interim-PET (VIF = 1.11), EoT PET (VIF = 1.17), nodal below diaphragm location type (VIF = 1.52), and extranodal location type (VIF = 1.39). Coefficient values in green have a p-value < 0.05. The rightmost columns for each method block report model-fit statistics (log-likelihood, AIC, BIC, Nagelkerke R² and Chi-square goodness-of-fit).

In summary, an increase in SUVpeak was associated with both under- and over-segmentation when using A50peak and 41%max methods. Contrarily, over-segmentation by these methods was less likely when TBRpeak and SUVbg values increased. The absolute threshold methods SUV2.5 and SUV4.0 demonstrated a comparable association pattern, in which an upswing in SUVpeak, TBRpeak, and SUVbg values reduced the risk of under-segmentation. However, with a lower threshold than SUV4.0, SUV2.5 was more susceptible to generating over-segmented VOIs as SUVpeak and SUVbg values increased. Compared to A50peak, 41%max, and SUV2.5, higher TBRpeak values were negatively associated with over-segmentation when using MV2, while higher SUVpeak values increased the risk of over-segmentation. MV3 showed broadly similar association patterns to MV2, with SUVpeak positively associated with both under- and over-segmentation and TBRpeak/SUVbg generally negatively associated with over-segmentation, although the effect sizes and overall model fit were smaller for MV3.

| **Method** | **Rating** | **SUVpeak** | **TBRpeak** | **SUVbg** | **Interim PET** | **EoT PET** | **Nodal below D** | **Extranodal** | **Model fit indicators** | |
| --- | --- | --- | --- | --- | --- | --- | --- | --- | --- | --- |
| **A50peak** | 1 | 0,42 | -0,99 | -1,19 | 0,28 | -1,72 | 1,11 | 1,02 | LogLikelihood | -562,52 |
| **A50peak** | 2 | 0,21 | 0,02 | -0,15 | -0,07 | -0,05 | -0,18 | 0,46 | AIC | 1189,05 |
| **A50peak** | 4 | 0,20 | -0,57 | -1,75 | 0,14 | 0,18 | -0,41 | -0,22 | BIC | 1329,64 |
| **A50peak** | 5 | 0,49 | -2,40 | -3,01 | -0,11 | -0,25 | 0,38 | 0,87 | NagelkerkeR2 | 0,24 |
|  |  |  |  |  |  |  |  |  | ChiSqGOF | 363,81 |
| **41%max** | 1 | -0,01 | 0,02 | 0,44 | 0,64 | -1,34 | 1,90 | 2,13 | LogLikelihood | -598,35 |
| **41%max** | 2 | 0,12 | 0,13 | 0,02 | 0,47 | 0,93 | -0,70 | -0,28 | AIC | 1260,70 |
| **41%max** | 4 | 0,28 | -0,75 | -1,14 | 1,23 | 0,75 | -0,50 | -0,23 | BIC | 1401,29 |
| **41%max** | 5 | 0,82 | -2,93 | -3,04 | 0,92 | 0,07 | 0,27 | 0,50 | NagelkerkeR2 | 0,23 |
|  |  |  |  |  |  |  |  |  | ChiSqGOF | 363,57 |
| **SUV2.5** | 1 | -3,77 | -0,43 | -2,59 | -9,80 | 2,46 | -8,18 | 0,26 | LogLikelihood | -460,90 |
| **SUV2.5** | 2 | -0,67 | -0,47 | -0,91 | -0,49 | -1,33 | 0,47 | 0,13 | AIC | 985,80 |
| **SUV2.5** | 4 | 0,63 | -0,01 | 2,29 | -0,56 | 0,77 | 0,29 | 0,61 | BIC | 1126,39 |
| **SUV2.5** | 5 | 0,67 | -0,28 | 2,10 | -0,48 | -0,21 | 0,61 | 1,04 | NagelkerkeR2 | 0,36 |
|  |  |  |  |  |  |  |  |  | ChiSqGOF | 524,11 |
| **SUV4.0** | 1 | -1,09 | -0,37 | -2,71 | 1,82 | 0,18 | -1,16 | -0,39 | LogLikelihood | -372,59 |
| **SUV4.0** | 2 | -0,38 | -0,18 | -2,27 | 0,36 | -0,22 | -0,73 | -0,64 | AIC | 793,18 |
| **SUV4.0** | 4 | 0,15 | -0,26 | -0,71 | -0,62 | -1,68 | 1,26 | 0,77 | BIC | 898,63 |
|  |  |  |  |  |  |  |  |  | NagelkerkeR2 | 0,36 |
|  |  |  |  |  |  |  |  |  | ChiSqGOF | 410,69 |
| **MV2** | 1 | 0,07 | -0,21 | -1,14 | 12,89 | -2,89 | 7,46 | 7,79 | LogLikelihood | -361,64 |
| **MV2** | 2 | 0,26 | -0,25 | -4,13 | -12,34 | 1,80 | -15,77 | -0,14 | AIC | 787,28 |
| **MV2** | 4 | 0,09 | -0,51 | -0,53 | 0,69 | 0,82 | -0,18 | 0,01 | BIC | 927,87 |
| **MV2** | 5 | 0,61 | -2,51 | -2,19 | 0,83 | 0,16 | 0,57 | 0,82 | NagelkerkeR2 | 0,23 |
|  |  |  |  |  |  |  |  |  | ChiSqGOF | 217,00 |
| **MV3** | 1 | 0,48 | -1,08 | -2,38 | -1,00 | -0,13 | 0,37 | 1,39 | LogLikelihood | -522,75 |
| **MV3** | 2 | 0,15 | 0,03 | -0,23 | 0,08 | -0,23 | -0,27 | -0,11 | AIC | 1109,50 |
| **MV3** | 4 | 0,30 | -0,65 | -0,97 | 0,20 | 0,55 | -0,59 | 0,28 | BIC | 1250,10 |
| **MV3** | 5 | 0,26 | -1,52 | -0,95 | -0,51 | -0,82 | 0,78 | 1,49 | NagelkerkeR2 | 0,15 |
|  |  |  |  |  |  |  |  |  | ChiSqGOF | 190,33 |
