# Supplementary material for: A Lesion-adaptive Segmentation Approach for Tumor Delineation on FDG PET/CT in Diffuse Large B-cell Lymphoma Patients
Source: Eur J Nucl Med Mol Imaging. 2026 Feb 14;53(6):4175–85. doi: 10.1007/s00259-026-07768-8 (PMC13121395; doi:10.1007/s00259-026-07768-8)
Supplement: Supplementary file 7 — (DOCX 25.9 KB) [file 259_2026_7768_MOESM5_ESM.docx]

**Supplemental Table 3a**. Machine-learning models and corresponding hyperparameter grids used for 5-fold cross-validated grid search within the 80% training sets.

For each machine-learning (ML) algorithm, an extensive grid search was performed over the hyperparameter ranges listed in this table. In every experiment, 80% of the lesions were used for model development and 20% for testing. Within each 80% training set, 5-fold cross-validation was used to identify the optimal hyperparameter combination. This procedure was repeated for 50 independent random 80%/20% train–test splits. In each split, the model with the best cross-validated performance was retrained on the full 80% training set and then evaluated on the corresponding 20% test set. Mean accuracies and standard deviations across these 50 test sets are reported in Supplementary Table 3b.

**Supplemental Table 3b**. Method-selection strategies and performance of machine-learning classifiers.

Each trained classifier outputs, for every lesion and segmentation method, a predicted probability of achieving a rating-3 segmentation. Several families of method-selection strategies were evaluated using these probabilities:

- Highest-probability selection (primary strategy): for each lesion, the segmentation method with the highest predicted probability of rating 3 was selected, treating all candidate methods symmetrically and imposing no a priori preference ordering.

- Hierarchical selection strategies: schemes in which one or more pre-specified methods (e.g., SUV4.0, SUV2.5, A50P, MV2, MV3) were given priority if their predicted probability exceeded a predefined threshold (e.g., 0.5, 0.6 or 0.8); otherwise, selection defaulted to another method (e.g., the overall highest-probability method or a predefined alternative).

- Restricted method sets: strategies restricted to subsets of three to five methods, most notably {SUV4.0, MV2, MV3}, using either the highest-probability or the hierarchical rules within this reduced output set.

- Stacked ensemble approaches: stacked ensembles in which a second-level meta-classifier (Logistic Regression, Decision Tree or Random Forest) was trained on the vectors of base-model probabilities (one probability per segmentation method) for each lesion, with or without inclusion of the original SUVpeak, TBRpeak and SUVbg features.

The table lists the 40 best classifier-strategy combinations ranked by mean accuracy across the 50 independent 80%/20% train-test splits. Mean accuracies varied only modestly (0.77–0.83). The highest value was obtained with a Support Vector Machine using the simple highest-probability rule across five methods (A50P, 41%max, SUV2.5, SUV4.0, MV2), with a mean accuracy of 0.83 (SD 0.03). Several other highest-probability configurations using Random Forest, Logistic Regression, XGBoost or LightGBM, either on the full set of methods or on restricted sets, achieved accuracies around 0.81–0.82. Strategies restricted to SUV4.0, MV2 and MV3 with the highest-probability rule yielded accuracies of 0.816–0.819, indicating that limiting the output set to these three methods has little impact on performance. In contrast, hierarchical schemes and stacked ensembles generally achieved accuracies of 0.80 or lower and did not outperform the simpler highest-probability strategies.

**Supplemental Table 3c**. Correlation between ML-selected segmentations and reference MTV/TMTV.

| **Timing** | **Method** | **Lesion-level ρ (per-lesion MTV vs reference)** | **Patient-level ρ (TMTV vs reference)** |
| --- | --- | --- | --- |
| Overall | Decision Tree | 0.77 | 0.89 |
| Overall | Random Forest | 0.82 | 0.91 |
| Overall | LightGBM | 0.81 | 0.88 |
| Overall | XGBoost | 0.82 | 0.86 |
| Overall | Logistic Regression | 0.81 | 0.94 |
| Overall | Support Vector Machine. | 0.81 | 0.89 |

Spearman rank correlation coefficients (ρ) between metabolic tumour volumes (MTV) derived from ML-selected segmentation methods and the reference segmentations are shown at lesion level (per-lesion MTV) and patient level (total MTV, TMTV), aggregated over all treatment time points. All ML approaches yielded relatively high correlations at patient level (ρ 0.86–0.94) and slightly lower correlations at lesion level (ρ 0.77–0.82). Logistic Regression showed the highest patient-level correlation with the reference TMTV (ρ = 0.94).
