# Supplementary material for: A Lesion-adaptive Segmentation Approach for Tumor Delineation on FDG PET/CT in Diffuse Large B-cell Lymphoma Patients
Source: Eur J Nucl Med Mol Imaging. 2026 Feb 14;53(6):4175–85. doi: 10.1007/s00259-026-07768-8 (PMC13121395; doi:10.1007/s00259-026-07768-8)
Supplement: Supplementary file 8 — (DOCX 16.3 KB) [file 259_2026_7768_MOESM6_ESM.docx]

| **Decision Rule variants** | **SUVpeak Threshold** | **TBRpeak Treshold** | **SUVbg Threshold** | **Mean Accuracy** | **Standard Deviation** |
| --- | --- | --- | --- | --- | --- |
| If SUVpeak > threshold: **SUV4**. Else, If SUVbg > threshold: **MV3**, otherwise: **MV2** | 8,5 | - | 0,8 | 0,823 | 0,029 |
| If SUVpeak > threshold: **SUV4**. Else, If SUVbg > threshold: **MV3**, otherwise: **MV2** | 8 | - | 0,8 | 0,823 | 0,029 |
| If SUVpeak > threshold: **SUV4**. Else, If SUVbg > threshold: **MV3**, otherwise: **MV2** | 7,5 | - | 0,8 | 0,823 | 0,029 |
| If SUVpeak > threshold: **SUV4**. Else, If SUVbg > threshold: **MV3**, otherwise: **MV2** | 7 | - | 0,8 | 0,823 | 0,029 |
| If SUVpeak > threshold: **SUV4**. Else, If SUVbg > threshold: **MV3**, otherwise: **MV2** | 6,5 | - | 0,8 | 0,821 | 0,030 |
| If SUVpeak > threshold: **SUV4**. Else, If SUVbg > threshold: **MV3**, otherwise: **MV2** | 8 | - | 0,9 | 0,819 | 0,031 |
| If SUVpeak > threshold: **SUV4**. Else, If SUVbg > threshold: **MV3**, otherwise: **MV2** | 7,5 | - | 0,9 | 0,819 | 0,031 |
| If SUVpeak > threshold: **SUV4**. Else, If SUVbg > threshold: **MV3**, otherwise: **MV2** | 7 | - | 0,9 | 0,819 | 0,031 |
| If SUVpeak > threshold: **SUV4**. Else, If SUVbg > threshold: **MV3**, otherwise: **MV2** | 6,5 | - | 0,9 | 0,819 | 0,031 |
| If SUVpeak > threshold: **SUV4**. Else, If SUVbg > threshold: **MV3**, otherwise: **MV2** | 6 | - | 0,8 | 0,819 | 0,030 |

**Supplemental Table 4.** The ten best-performing decision rule variants include all the same methods (SUV4, MV3, MV2) in the same decision order and use the same uptake metrics (SUVpeak, SUVbg).
